# Critical illness induces nutrient-independent adipogenesis and accumulation of alternatively activated tissue macrophages

**DOI:** 10.1186/cc12887

**Published:** 2013-09-10

**Authors:** Mirna Bastos Marques, Sarah Vander Perre, Annelies Aertgeerts, Sarah Derde, Fabian Güiza, Michael P Casaer, Greet Hermans, Greet Van den Berghe, Lies Langouche

**Affiliations:** 1 Laboratory of Intensive Care Medicine, Department of Cellular and Molecular Medicine, KU Leuven, Herestraat 49 bus 503, B-3000 Leuven, Belgium

## Abstract

**Introduction:**

We previously reported that in artificially-fed critically ill patients, adipose tissue reveals an increase in small adipocytes and accumulation of M2-macrophages. We hypothesized that nutrient-independent factors of critical illness explain these findings, and that the M2-macrophage accumulation may not be limited to adipose tissue.

**Methods:**

In a long-term cecal ligation and puncture (CLP) mouse model of sepsis, we compared the effect of parenteral nutrition (CLP-fed, *n *= 13) with nutrient restriction (CLP-restricted, *n *= 11) on body composition, adipocyte size and macrophage accumulation in adipose tissue, liver and lungs. Fed healthy mice (*n *= 11) were studied as controls. In a human study, *in vivo *adipose tissue biopsies were studied from ICU patients (*n *= 40) enrolled in a randomized control trial which compared early initiation of parenteral nutrition (PN) *versus *tolerating nutrient restriction during the first week of ICU stay. Adipose tissue morphology was compared with healthy human controls (*n *= 13).

**Results:**

Irrespective of nutritional intake, critically ill mice lost weight, fat and fat-free mass. Adipocyte number, proliferation marker Proliferating Cell Nuclear Antigen (PCNA) and adipogenic markers PPARγ and CCAAT/enhancer binding protein-β (C/EBPβ) increased with illness, irrespective of nutritional intake. M2-macrophage accumulation was observed in adipose tissue, liver and lungs of critically ill mice. Macrophage M2-markers correlated with CCL2 expression. In adipose tissue biopsies of critically ill patients, increased adipogenic markers and M2 macrophage accumulation were present irrespective of nutritional intake.

**Conclusions:**

Adipogenesis and accumulation of tissue M2-macrophages are hallmarks of prolonged critical illness, irrespective of nutritional management. During critical illness, M2-macrophages accumulate not only in adipose tissue, but also in the liver and lungs.

## Introduction

Several observational studies have shown lower mortality in overweight and obese versus lean critically ill patients, an association known as the 'obesity paradox' [1-4]. We previously documented increased formation of small adipocytes during critical illness with increased glucose and lipid storage properties [5,6]. These changes might be beneficial during critical illness as newly-formed, small adipocytes with augmented insulin sensitivity and lipid-storage capacity might prevent excess circulating lipids and glucose [7]. In addition, we demonstrated a remarkable macrophage accumulation in adipose tissue of critically ill patients with a predominant M2-phenotype [6]. Increased insulin sensitivity and β-oxidation of free fatty acids (FFA), but also phagocytosis of infectious microorganisms, production of anti-inflammatory and tissue-healing factors are potential beneficial features of M2-macrophages during critical illness (CI) [8].

These previous observations were based on biopsies from prolonged severely ill patients who had received parenteral nutrition (PN) throughout the ICU stay. The observed changes in adipose tissue (AT) might thus be related to the administration of PN or to nutrition-independent factors. In health, excessive caloric intake promotes hypertrophy and hyperplasia of adipocytes, and accumulation of M1-macrophages [4,9]. Macrophage accumulation could also be evoked by catecholamines and hypoxia, factors accepted to play a role in the pathophysiology of sepsis [10,11]. It is thus plausible that other tissues involved in innate immunity could also display macrophage accumulation during CI.

We hypothesized that nutrition-independent factors evoke the morphological changes in AT during CI. We, therefore, studied the impact of PN administration, as compared with nutrient restriction, on body composition, adipogenesis and macrophage accumulation in AT, liver and lungs in an antibiotic-treated fluid-resuscitated mouse model of sepsis. In addition, we studied adipogenesis and macrophage accumulation in subcutaneous AT biopsies harvested *in vivo *after one week of critical illness from human patients enrolled in a randomized, controlled trial (RCT) in which early initiation of PN was compared with tolerating nutrient restriction in the first seven days of ICU stay [12].

## Materials and methods

### Set-up of the experimental animal model

**Figure 1:**
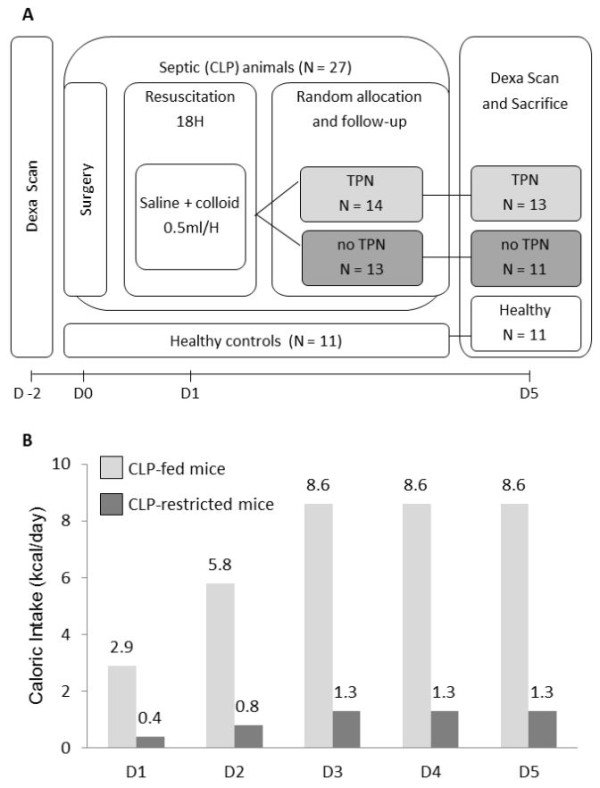
**Experimental set-up of the animal work**. (**A**) Timeline and mortality of the model. (**B**) Caloric intake: all mice received total parenteral nutrition (TPN) or saline + glucose through an indwelling jugular catheter. The administered fluid/TPN regimen was stepwise increased from 0.1 ml/H to the maximum sustained dose of 0.3 ml/H.

The protocol for this study was approved by the Institutional Ethical Committee for Animal Experimentation (project number P051/2010). Male, 24-week old, C57BL/6J mice (Janvier SAS, Chassal, France) were anesthetized with an intraperitoneal (i.p.) injection of ketamine (3 mg, Univet, Heusden-Zolder, Belgium) and xylazine (0.4 mg, VMD, Brussels, Belgium) and submitted to dual-energy-X-ray-absorptiometry (DEXA) to identify body composition (Lunar PIXImus™, GE Medical-Lunar, Madison, WI, USA) (Figure 1). After a 48-hour recovery, animals were again anaesthetized by i.p. injection of ketamine and xylazine. Animals were placed on a heating pad maintained at 25°C. Spontaneously breathing mice were continuously inhaling 1 to 2% isoflurane-vet (Schering-Plough, Hull, UK) with oxygen (2L/min) through a facial mask. The left central jugular vein was dissected under microscopy and a sterilized Renathane catheter (model MRE025; Braintree Scientific, Braintree, MA, USA) was inserted. The catheter was tunneled to the back, attached to a single-channel-fluid-swivel (model 375/25; Instech Laboratories, Plymouth Meeting, PA, USA) allowing free movement and continuous perfusion according to the resuscitation scheme (Figure 1). Sepsis was produced by a 50% ligation of the cecum (at half the distance between the distal pole and the base of the cecum) with a 3.0 silk suture (Mersilk, Ethicon, Johnson & Johnson, St.-Stevens-Woluwe, Belgium), and a single puncture through-and-through with a 20-gauge needle. The cecum was gently squeezed to extrude feces and returned to its original position [13-15]. Surgical wounds were closed with 7.0 silk sutures (Mersilk, Ethicon, Johnson & Johnson, St.-Stevens-Woluwe, Belgium). Post-operative analgesia was provided by local infiltration of the surgical wounds with ropivacaine (1%, AstraZeneca, Brussels, Belgium) and subcutaneous (sc) administration of buprenorphine (9 μg, Schering-Plough, UK). Mice received the broad-spectrum antibiotic Imipenem (0.25 mg sc, Meropenem, AstraZeneca, Brixham, UK) and continuous intravenous fluid-resuscitation (NaCl_0.45%_/glucose_2.5% _+ 6% hydroxyethyl starch (Voluven, Fresenius Klabi, Schelle, Belgium)) in a 4:1 proportion at 0.5 ml/H for the first 18 hours [16]. Afterwards, animals were randomly allocated to the "restricted" or "fed" protocol. Animals in the 'CLP-restricted' protocol (*n *= 13) received progressive increments of NaCl_0.45%_/glucose_50% _(0.4 to 1.3 Kcal/day). Animals in the 'CLP-fed' protocol (*n *= 14) received increments of total parenteral nutrition (TPN) (consisting of 29% kcal from lipids, 14% kcal from amino acids, and 57% kcal from glucose) from 2.9 to 8.6 Kcal/day (Clinomel N7, Baxter, Braine-l'Alleud, Belgium). Healthy controls (*n *= 11) received 5 g of standard chow daily (13 kcal/24 h). A nutrient-restricted healthy group (pair-fed for the CLP-restricted group) was unfeasible as the restricted nutrient intake was lethal for healthy mice. Every 12 hours septic mice received sc 0.25 mg Imipenem and 9 to 12 μg buprenorphine. Buprenorphine was chosen because it shares clinical attributes of standard opioid analgesics but evokes less respiratory depression and minor immune suppression in mice models [17]. Post-operative pain was regularly assessed by the Mice Grimace Scale [18]. All mice were kept normoglycemic (80 to 120 mg/dl) with iv infusion of insulin (Actrapid Penfill, Novo Nordisk, Bagsvaerd, Denmark) using an in-house developed protocol. To avoid hypothermia, the cage temperature was kept at 27 ± 1°C with heating lamps. Animals that lost their catheter were excluded from the study. After five days, surviving animals were submitted to a second DEXA-scan, and sacrificed by decapitation. Due to technical problems, not all mice were scanned twice. Blood was collected from the trunk, and organs were removed. AT was removed from the inguinal, epididymal and peri-renal sites. All tissue samples were snap-frozen in liquid nitrogen and stored at -80°C until analysis. Due to the small size of AT biopsies, not all biopsies could be analyzed for both immunocytochemistry and RNA analysis.

### Human study

Patients were enrolled in a large RCT (*n *= 4,640) to compare early *versus *late initiation of parenteral nutrition to supplement failing enteral feeding during the first week of CI. Details of the study were previously published [19]. The protocol and consent forms were approved by the Institutional Ethical Review Board (ML4190). During the randomized control trial, subcutaneous biopsies were collected from 188 patients. To avoid bias of prior-to-illness body mass index (BMI)-related changes in the AT, only patients with a normal BMI (between 18 to 25 kg/m^2^) were selected for this study (*n *= 72). From these 72 patients, 20 early PN and 20 late PN patients, matched for admission characteristics and illness severity (Table 1), were compared with 13 healthy demographically-matched volunteers. On Day 8 ± 1 of the ICU stay, a subcutaneous *in vivo *AT biopsy was collected in the quadriceps region with a 5-mm-Bergström muscle-biopsy cannula according to the protocol described earlier [6]. The biopsy specimens were snap frozen in liquid nitrogen and stored at -80°C.

**Table 1:** Baseline and outcome variables of studied critically ill patients and healthy volunteers

	Early PN patients *N *= 20	Late PN patients *N *= 20	*P-value *Early *vs*. Late	Healthy Controls *N *= 13	*P-value *Ill *vs*. Healthy
*Demographic data*					
Male sex - no. (%)	15 (75)	14 (70)	*0.7*	8 (62)	*0.6*
Age - yr (mean ± SE)	60 ± 2	63 ± 3	*0.1*	60 ± 3	*0.4*
BMI - Kg/m^2 ^(mean ± SE)	23.1 ± 0.9	23.2 ± 1.2	*0.7*	23.9 ± 1.3	*0.1*
					
*Comorbidity parameters*					
Malignancy no. (%)	6 (30)	5 (25)	*0.7*		
Diabetes no. (%)	3 (15)	1 (5)	*0.3*		
NRS score - median [IQR]	4 [3 to 7]	4 [3 to 6]	*0.4*		
APACHE II SCORE - median [IQR]	30 [21 to 47]	33 [23 to 42]	*0.6*		
Reason for admission - no. (%)			*0.7*		
Complicated Surgery/Trauma	11	10			
Medical	5	7			
Transplant	3	2			
Cardiac surgery	1	1			
					
*Outcome variables*					
New infection - no (%)	14 (70)	15 (75)	*0.7*		
Duration of ICU stay - median [IQR]	15 [10 to 33]	18 [10 to 40]	*0.7*		
ICU non-survivors - no (%)	3 (15)	4 (20)	*0.7*		

### Triglyceride content in liver and skeletal muscle

Triglyceride content of tissue was determined with a colorimetric commercial assay (triglyceride quantification kit, Abcam, Cambridge, UK).

### Immunohistochemistry

Paraformaldehyde-fixed paraffin sections were stained with F4/80Ab (general anti-macrophage antibody (Ab) for mice (Abcam)), CD163Ab (Santa Cruz Biotechnology, Santa Cruz, CA, USA), and Peroxisome Proliferator-Activated Receptor Gamma (PPARγ)-Ab (Abcam). For human samples, the general macrophage marker CD68 was used (Dako, Glostrup, Denmark). Macrophage accumulation in AT sections was evaluated in a blinded fashion by two observers on a Leica DM3000 Microscope (Wetzlar, Germany). Macrophage staining in liver and lungs, PPARγ staining in AT and adipocyte size were quantified on digital microscopical images with in-house designed algorithms developed in Matlab 7.4.0 and Image Processing Toolbox (Matworks, Natick, MA, USA) as described earlier [20]. The estimated proportion of adipocytes was calculated by dividing total end weight of body fat (second DEXA quantification) by mean adipocyte size. This calculation was then plotted proportionally to the mean estimated number of adipocytes of the healthy mice [21].

### Gene expression

Messenger RNA was isolated and cDNA quantified in real time as previously described [22]. Data are expressed normalized to GAPDH or HPRT as fold-change of the mean of the control group. For the quantification of Proliferating Cell Nuclear Antigen (PCNA), CCAAT/enhancer binding protein-β (C/EBPβ), hormone sensitive lipase (HSL), tumor necrosis factor-α (TNFα), inducible nitric oxide synthase (iNOS), Arginase-1, interleukin 10 (IL-10), chemokine (C-C motif) ligand 2 (CCL2), and CD-163 messenger RNA, commercial gene expression assays (TaqMan, Life Technologies, Gent, Belgium) were used.

### Statistics

The following predefined statistical plan was used: (1) to assess the impact of illness, the fed healthy controls were compared with the CLP-fed; (2) to assess the impact of feeding during illness, the CLP-fed were compared with the CLP-restricted. Unpaired t-test was used for normally distributed data; the non-parametric Mann-Whitney u test for non-normally distributed data. Exponential gene expression data were log transformed. For comparison of proportions, the X-square test was used. The significance of correlations was assessed by calculation of the Pearson (r) correlation coefficient. Statistical significance was considered when *P*-values ≤ 0.05.

## Results

### Mouse study - impact of PN during CI on body composition, adipogenesis and lipolysis

**Figure 2:**
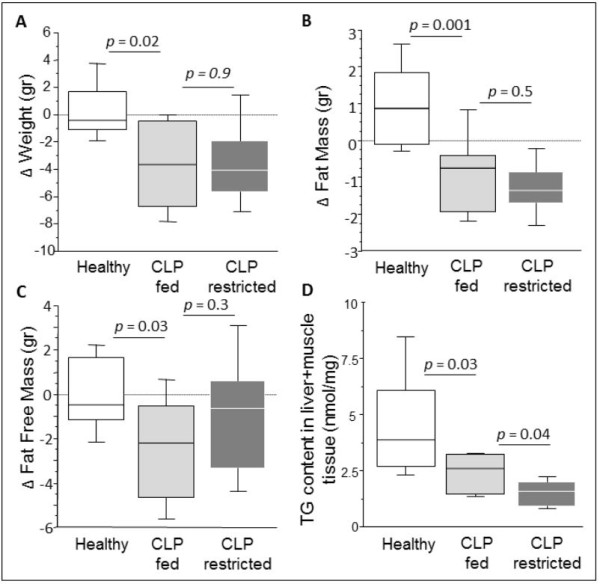
**Changes in mouse body composition over five days of sepsis**. The box plots represent the changes from baseline to Day 5 (referred to as "Δ") of total body weight (**A**), total body fat mass (**B**), total body fat-free mass (**C**) and triglyceride content in non-adipose tissues, liver and muscle (**D**). Data boxes present median and IQR; whiskers represent the 10^th ^and the 90^th ^percentiles. White boxes present healthy controls (*n *= 8), light gray boxes are critically ill mice allocated to the total parenteral nutrition (TPN) protocol (cecal ligation and puncture (CLP)-fed) (*n *= 8), and dark gray boxes are critically ill mice allocated to the nutrient restriction (CLP-restricted) protocol' (*n *= 8).

The beginning bodyweight of the animals was 29.3 ± 0.4 g (mean ± SE), of which 4.2 ± 0.1 g were fat. Changes in body composition were quantified in eight animals per group. CLP-fed animals lost weight, total body fat mass and total body fat-free mass as compared with healthy animals (Figure 2A-C). The loss of fat mass in CLP-fed mice corresponded to a 26% reduction in total body fat mass (from mean 4.1 ± 0.1 g to 3.4 ± 0.2g). CLP-fed and CLP-restricted animals showed a comparable reduction in lost weight, fat mass and fat-free mass.

Part of the fat was lost from ectopic sites, evidenced by lowered triglyceride content in liver and skeletal muscle in CLP-fed compared to healthy mice. Reduction of triglyceride content from liver and skeletal muscle was further accentuated in CLP-restricted compared to CLP-fed animals (Figure 2D). Total body fat mass correlated with triglyceride content in liver and muscle (R = 0.625, *P *= 0.01).

**Figure 3:**
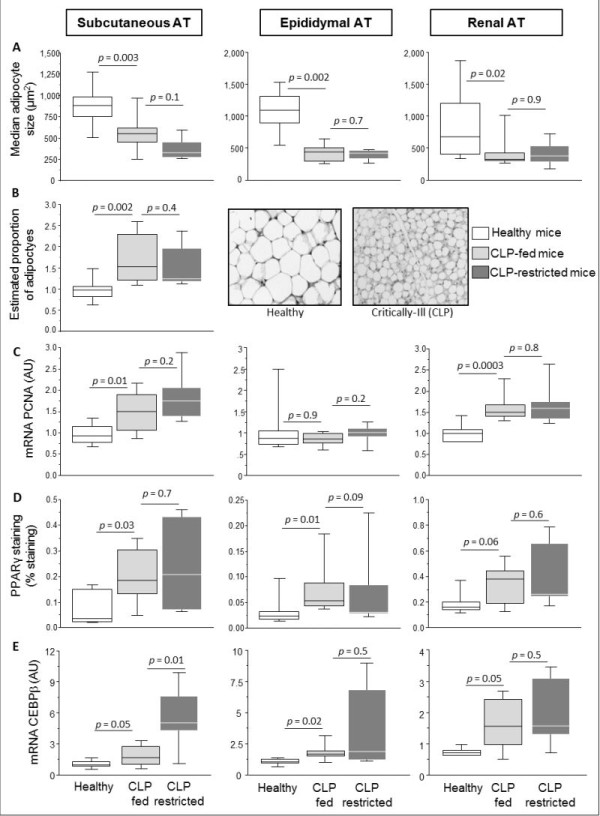
**Adipogenic markers in subcutaneous and visceral adipose tissue of septic mice**. The box plots represent median adipocyte size (**A**), estimated proportion of adipocytes and representative microscopic adipose tissue images of a healthy and a cecal ligation and puncture (CLP)-fed mouse (**B**), Proliferating Cell Nuclear Antigen (PCNA) gene expression (**C**), percentage stained area for peroxisome proliferator-activated receptor γ (PPARγ) (**D**), mRNA gene expression of CCAAT/enhancer binding protein-β (C/EBPβ) (**E**). AT is adipose tissue, A.U. is arbitrary units. Data boxes present median and IQR; whiskers represent the 10^th ^and the 90^th ^percentiles. For immunocytochemistry analyses in A, B and D, White boxes present healthy controls (*n *= 10), light gray boxes are critically ill mice allocated to the total parenteral nutrition (TPN) protocol (CLP-fed) (*n *= 6), and dark gray boxes are critically ill mice allocated to the nutrient restriction protocol (CLP-restricted) (*n *= 8). For gene expression analyses, the number of animals are healthy controls (*n *= 8), CLP-fed (*n *= 7), CLP-restricted (*n *= 6).

Adipocytes of CLP-fed mice were smaller than in healthy controls in subcutaneous (46% smaller), epididymal (62% smaller) and renal AT (51% smaller) (Figure 3A). CLP-restricted mice showed a comparable reduction in adipocyte size as CLP-fed mice. Because smaller adipocytes could theoretically be explained by more newly formed small adipocytes (adipogenesis), or by adipocytes releasing stored triglycerides (lipolysis), we quantified markers for both processes. To assess lipolysis, gene expression of HSL, the rate-limiting enzyme in the release of stored triglycerides, was quantified. HSL gene expression was lower in CLP-fed than in healthy animals in subcutaneous AT (median (IQR) 1.3 (0.8 to 1.5) to 0.4 (0.4 to 0.5) Arbitrary Units (AU), *P *= 0.05) and renal AT (1.0 (0.9 to 1.2) to 0.6 (0.6 to 0.7) AU, *P *= 0.005). In the epididymal AT, HSL gene expression tended (*P *= 0.1) to be lower in CLP-fed than in healthy mice. There was a significant further reduction of HSL gene expression in visceral (*P *= 0.004), but not in subcutaneous AT (*P *= 0.7) of CLP-restricted compared to CLP-fed mice.

To assess adipogenesis, the number of adipocytes was estimated by dividing total body fat mass by adipocyte size: CLP-fed mice displayed 50% higher number of adipocytes compared to healthy mice (Figure 3B). CLP-fed and CLP-restricted mice showed a comparable increased number of adipocytes. Gene expression of the proliferation marker PCNA was up-regulated in CLP-fed mice compared to healthy controls in subcutaneous and renal AT (not in epidydimal AT). CLP-fed and CLP-restricted mice showed a comparable PCNA gene expression (Figure 3C). Immunostaining of the adipogenic transcription factor PPARγ was significantly higher in CLP-fed compared to healthy mice in all AT depots. CLP-fed and CLP-restricted mice showed comparable PPARγ immunostaining (Figure 3D). C/EBPβ expression was higher in CLP-fed than in healthy animals in all AT depots. In subcutaneous AT, CLP-restricted mice showed a further increase in C/EBPβ gene expression when compared to CLP-fed mice (Figure 3E). In visceral AT, CLP-fed and CLP-restricted mice showed comparable increase in C/EBPβ gene expression.

### Mouse study: impact of PN during CI on tissue macrophages

**Figure 4:**
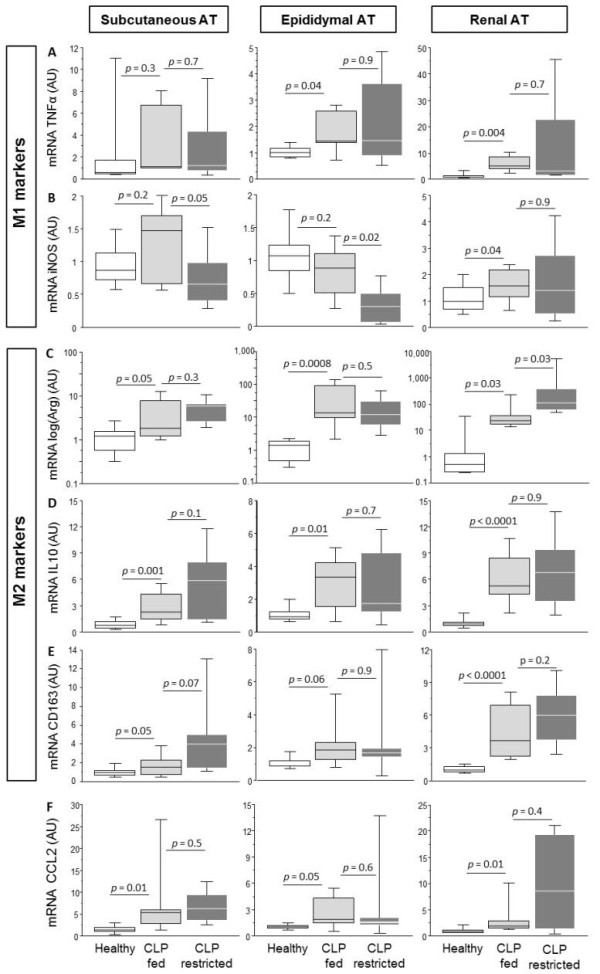
**M1/M2 phenotyping of the macrophages in subcutaneous and visceral adipose tissue of septic mice**. Gene expression of the M1-type macrophage marker tumor necrosis factor alpha (TNFα) (**A**), and inducible nitrous oxide synthase (iNOS) (**B**), Gene expression of the M2-type macrophage markers Arginase 1 (**C**), interleukin (IL)10 (**D**), and CD163 (**E**). Gene expression of the chemoattractant factor chemokine ligand 2 (CCL2) (**F**). AT is adipose tissue, A.U. is arbitrary units. Data boxes present median and IQR; whiskers represent the 10^th ^and the 90^th ^percentiles. White boxes present healthy controls (*n *= 8), light gray boxes are critically ill mice allocated to the total parenteral nutrition (TPN) protocol (cecal ligation and puncture (CLP)-fed) (*n *= 7), and dark gray boxes are critically ill mice allocated to the nutrient restriction protocol (CLP-restricted) (*n *= 6).

Immunostaining revealed that macrophages accumulated in all AT depots of CLP-fed animals compared with healthy animals. There was comparable accumulation of macrophages in CLP-fed and CLP-restricted animals (Table 2). Macrophages can polarize to either an M1- or an M2-phenotype, each expressing distinct markers. We quantified gene expression of TNFα and iNOS as M1-markers; and arginase-1, IL10 and CD163 as M2-markers. In subcutaneous AT of CLP-fed mice, gene expression of TNFα and iNOS was similar to healthy mice (Figure 4A, B). In contrast, the M2-markers arginase-1, IL-10 and CD163 were markedly up-regulated in subcutaneous AT of CLP-fed mice compared to healthy mice. In visceral AT, TNFα (renal and epididymal) and iNOS (renal) gene expressions were increased up to two-fold in CLP-fed compared to healthy mice (Figure 4A, B). In contrast, gene expression of the M2-marker arginase-1 increased more than 10-fold in CLP-fed compared to healthy mice.

**Table 2:** Macrophage accumulation in adipose tissue of septic mice

Macrophage Accumulation	Score 0	Score 1	Score 2	*P-values*
**Subcutaneous AT**				
Healthy (*n *= 8)	100%	-	-	
CLP-fed (*n *= 6)	-	83%	17%	Ill *vs *Healthy: *P *= 0.0009
CLP-restricted (*n *= 6)	-	33%	67%	Fed *vs *Restricted: *P *= 0.6
				
**Epidydimal AT**				
Healthy (*n *= 6)	100%	-	-	
CLP-fed (*n *= 6)	-	33%	67%	Ill *vs *Healthy: *P *= 0.01
CLP-restricted (*n *= 7)	-	28%	72%	Fed *vs *Restricted: *P *= 0.8
				
**Renal AT**				
Healthy (*n *= 11)	64%	27%	9%	
CLP-fed (*n *= 11)	-	55%	45%	Ill *vs *Healthy: *P *= 0.005
CLP-restricted (*n *= 5)	-	40%	60%	Fed *vs *Restricted: *P *= 0.6

Every sample received a score 0 (no positive cells visible), score 1 (intermediate number of positive cells), or 2 (high number of positive cells). AT, adipose tissue

Expression of M1 and M2 markers was comparable in CLP-fed and CLP-restricted mice in all AT depots (Figure 4). Only in subcutaneous and epididymal AT, iNOS was reduced and in renal AT, arginase expression was further increased in CLP-restricted compared to CLP-fed mice. Subcutaneous and visceral AT sections were immunostained for CD163. Positive M2 macrophage staining was present in all AT depots of CLP-fed and CLP-restricted mice.

**Figure 5:**
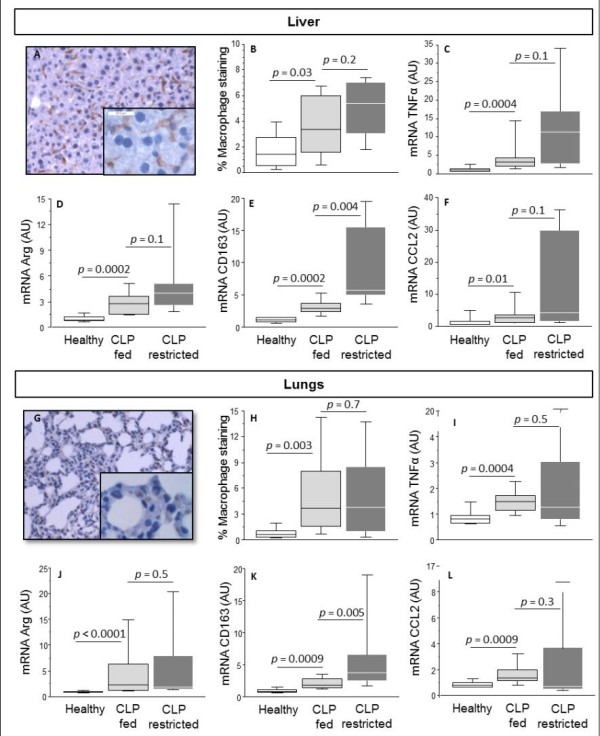
**Macrophage accumulation in the liver and lungs of septic mice**. The upper panel displays liver parameters, the bottom panel displays lung parameters. Representative microscopic image of tissue macrophage staining (**A, G**). Computerized quantification of macrophage immunostaining (**B, H**). Gene expression of the M1-type macrophage marker tumor necrosis factor alpha (TNFα) (**C, I**), and M2-markers Arginase-1 (**D, J**) and CD163 (**E, K**). Gene expression of the chemo-attractant factor chemokine ligand 2 (CCL2) (**F, L**). AU is arbitrary units. Data boxes present median and IQR; whiskers represent the 10^th ^and the 90^th ^percentiles. White boxes present healthy controls (*n *= 13), light gray boxes are critically ill mice allocated to the total parenteral nutrition (TPN) protocol (cecal ligation and puncture (CLP)-fed) (*n *= 11), and dark gray boxes are critically ill mice allocated to the restriction protocol (CLP-restricted) (*n *= 11).

Immuno-staining revealed that macrophages also accumulated in the liver and lungs of CLP-fed compared with healthy mice (Figure 5). This increased staining corresponded in the liver and lungs with an increased expression of both M1-marker TNFα and M2-markers arginase-1 and CD163. In the liver, macrophage immuno-staining correlated significantly with CD163 gene expression (R = 0.541, *P *= 0.002) and with TNFα gene expression (R = 0.405, *P *= 0.003). There was equal accumulation of macrophages in the liver and lungs of CLP-fed and CLP-restricted mice. Also, expression of M1-marker TNFα and M2-marker arginase-1 was comparable in CLP-fed and CLP-restricted mice. In contrast, expression of M2-marker CD163 was further increased in CLP-restricted when compared to CLP-fed mice (Figure 5).

As a possible trigger for the macrophage accumulation, we quantified gene expression of the chemo-attractant factor CCL2. The gene expression of CCL2 was higher in all AT depots, lungs and liver of CLP-fed when compared to healthy mice (Figures 4F and 5). The gene expression of CCL2 in CLP-fed and CLP-restricted mice was comparable in all AT depots, lungs and liver. CCL2 expression correlated positively with arginase expression (R = 0.566, *P *= 0.007 in inguinal AT, R = 0.912, *P *< 0.0001 in epidydimal AT, R = 0.598, *P *= 0.009 in renal AT). CCL2 gene expression correlated positively with CD163 gene expression in the liver (R = 0.424, *P *= 0.01) and lungs (R = 0.730, *P *< 0.0001).

### Human study: impact of PN during CI on adipose tissue morphology and gene expression

**Figure 6:**
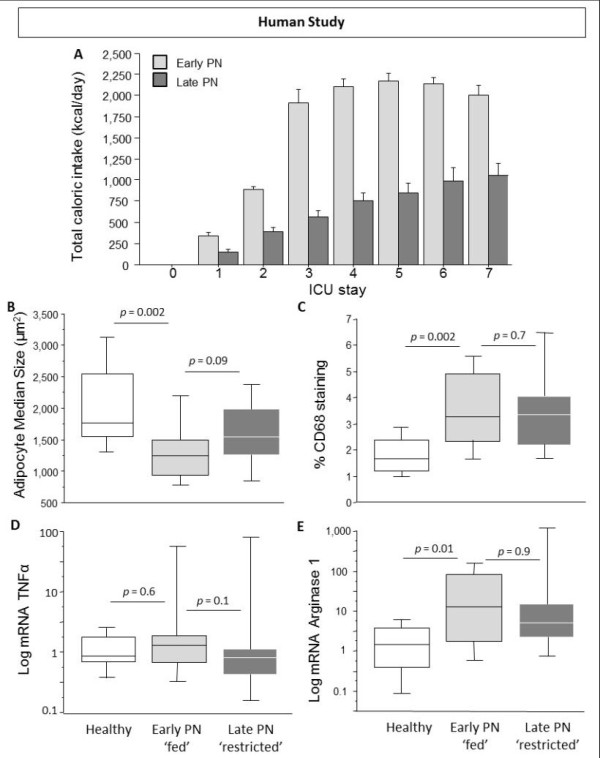
**Adipogenesis and macrophage accumulation in human *in vivo *subcutaneous adipose tissue biopsies**. Total caloric intake during first seven days in ICU (**A**), Median adipocyte size (**B**), Computerized quantification of macrophage immunostaining (**C**), Gene expression of the M1-type macrophage marker tumor necrosis factor alpha (TNFα) (**D**), Gene expression of the M2-type macrophage marker Arginase1 (**E**). AU is arbitrary units. Data boxes present median and IQR; whiskers represent the 10th and the 90th percentiles. White boxes present healthy volunteers (*n *= 13), light gray boxes are critically ill patients allocated to the early parenteral nutrition (PN) protocol (*n *= 20), and dark gray boxes are patients allocated to the late PN protocol (*n *= 20).

To assess the clinical relevance of our animal study observations, we additionally studied a matched set of 40 human AT biopsies collected *in *vivo during the EPaNIC trial [12]. Small needle biopsies were collected at Day 8, and compared with 13 biopsies from healthy volunteers. Total caloric intake was significantly higher in early-PN patients (*n *= 20) compared to late-PN (*n *= 20) patients during the first seven days in the ICU (Figure 6A).

Adipocyte size decreased in early PN 'fed' critically-ill patients compared to healthy controls (Figure 6B). Medium adipocyte size was equally reduced in early PN 'fed' patients and late PN 'restricted' patients. Immuno-staining revealed accumulation of macrophages in all biopsies of critically-ill patients (Figure 6C). Gene expression of TNFα was unaffected by illness, while arginase-1 gene expression was higher in early PN 'fed' critically-ill patients than in controls (Figure 6D, E). Gene expression of TNFα was equally up-regulated in early PN 'fed' and late PN 'restricted' patients.

It is known from the literature that glucocorticoids can both stimulate adipogenesis [23] and promote M2 polarization [24]. Nineteen of the 40 patients received corticosteroids during the study period. However, arginase expression (*P *= 0.25), TNFα expression (*P *= 0.47) and adipocyte size (*P *= 0.6) were not different between patients receiving or not receiving steroids. In contrast, patients receiving steroids during the study period, had a higher percentage of CD68 staining then patients not receiving steroids (*P *= 0.03).

## Discussion

Sepsis-induced critical illness in mice evoked nutrition-resistant loss of body weight, fat-free mass and fat mass, the latter partially explained by ectopic triglyceride loss. Concomitantly, critical illness increased adipogenesis resulting in an increased number of smaller adipocytes, irrespective of nutrition. Macrophages of a predominant M2-phenotype, accumulated in subcutaneous and visceral AT as well as in the liver and lungs of both fed and fasted septic mice. In the liver and lungs, fasting appeared to further enhance M2 over M1 polarization. In all tested tissues of the septic mouse model, increased expression of the chemotatic factor CCL2 correlated positively with macrophage accumulation. In the human study, we could confirm that increased adipogenesis and M2-macrophage accumulation in the adipose tissue was present in critically-ill patients, independent from parenteral nutrition administration.

Critical illness uniformly induces hypercatabolism, characterized by profound loss of lean tissue [25]. In humans, aggressive artificial nutrition was shown unable to prevent lean tissue wasting, whereas fat mass was preserved or even increased [26-28]. In our septic mouse model, we observed loss of both fat and fat-free mass after five days of critical illness in both CLP-fed and CLP-restricted mice. The 26% reduction in total body fat mass in mice may seem to contradict the available human data [26,28]. This discrepancy might be due to the length of our experimental animal set-up, as five days can be considered quite an extended time for mice. On the other hand, the methodology used in our experiment allowed the quantification of the total amount of fat in the body, including ectopic lipid depositions. Indeed, we demonstrated a reduction of triglyceride content in non-adipose tissues, such as liver and skeletal muscle, a reduction which was even more pronounced with nutrient restriction. Our observation of triglyceride reduction in liver does not contradict the known occurrence of fatty liver in critically ill patients, because our mouse model was well controlled for hyperglycemia, which is a major trigger for hepatic steatosis during critical illness [29,30].

Although some of the body fat mass was probably lost in the AT depots, the 26% reduction in total body fat mass contrasted sharply with the more than 40% reduction in median adipocyte size, indicating other factors involved in the decreased adipocyte size. Indeed, the observed reduction in adipocyte size could not be explained by lipolysis, as HSL expression was strongly reduced by illness and further reduced by fasting during illness. Instead, our data indicate that the increased number of small adipocytes resulted from increased adipogenesis. Indeed, the proliferation marker PCNA and nuclear transcription factors C/EBPβ and PPARγ were up-regulated, factors that are essential and sufficient to induce white adipocyte differentiation from stem cells [31]. The increase of the adipogenic markers was unaffected by nutrition, suggesting an illness-related trigger. A possible role of increased adipogenesis during critical illness would be to better deal with toxic metabolites, such as circulating triglycerides and glucose [32,33], preventing their accumulation in the liver and muscle. Newly-formed small adipocytes indeed have an increased capacity of storing triglycerides and glucose from the circulation [6,7,34]. Unexpectedly, fasting during CI further reduced lipolysis in visceral AT. This may suggest that during critical illness, fasting either does not signal the adipose tissue to release stored triglycerides or that increased adipogenesis evoked by illness overwhelms any lipolytic response.

The second important alteration we observed in AT during CI, again unaffected by nutrition, was the accumulation of macrophages. Similarly to the AT, the lungs and liver of critically ill mice showed a clear accumulation of macrophages, unaffected by nutrition. In all studied tissues, the markers for macrophage accumulation correlated strongly with CCL2, suggesting an orchestrated response to illness in innate immune-related organs. Cellular hypoxia and catecholamines, known to stimulate production and secretion of chemoattractant factors, may play a role in this process [10,35-37]. Additionally, recruited macrophages and adipocytes could further evoke catecholamine release inducing a vicious circle [38,39].

Depending on the trigger, tissue macrophages can polarize to the pro-inflammatory M1-state or to the anti-inflammatory M2-state [8]. The activation of M1 macrophages is a classical feature of cellular immune response to infection while the alternative activation of M2 macrophages plays a role in healing and repair. In this study, we observed a marked increase in M2 macrophages in AT, lungs and liver of critically ill mice and in AT of critically ill humans. In AT, M2 accumulation predominated compared to M1 markers, but in both liver and lungs of critically ill fed mice, M1 and M2 expression were increased concurrently. In the entire organism, macrophages are able to rapidly 'switch' from one phenotype to the other depending on the micro-environment [8]. Thus, a certain degree of overlap of phenotypes or even an 'in-between' phenotype can be expected [40,41]. From a metabolic point of view, the M1 to M2 'switch' during critical illness seems highly beneficial as M2 macrophages are able to efficiently engulf and β-oxidize FFA, and boost insulin sensitivity [41,42]. In this case, fasting might add some extra benefit as it is associated with a further M1 to M2 'switch' in the liver and lungs, thereby possibly protecting against ectopic lipid accumulation [43,44]. So, quite different from what is observed in obesity, during CI, M2 macrophages predominate. Altogether with the newly-formed adipocytes, M2 macrophages could prevent or reduce ectopic lipotoxicity [5,45]. We previously documented an increased expression of PPARγ as a possible trigger for the M2-phenotyping switch, and not the classically expected cytokines Il-4 or Il-13 [6]. In this study, we confirmed an increase in PPARγ, the master regulator of both adipogenesis and M1 to M2 switching [46-48]. Possible activators for PPARγ are circulating fatty acids and locally produced prostaglandins [48].

To assess the clinical relevance of the findings in the septic mouse model of CI, we studied human subcutaneous AT biopsies harvested from adult ICU patients, who participated in a RCT in which 'early initiation of parenteral nutrition' was compared with 'tolerating nutrient restriction' during the first week in ICU [12]. In these biopsies, taken *in vivo *after one week of illness in the ICU, we observed comparable alterations as in the mice. In critically ill humans, we could indeed demonstrate increased adipogenic markers and M2 macrophage accumulation, irrespective of nutrition.

The following limitations of the study should be highlighted: First, a sham group with surgical instrumentation required for the CLP model was not added. This was done as the key question was the impact of critical illness versus health and the role of nutrition versus nutrient restriction during critical illness. The surgical instrumentation and the treatment aspects of the model were considered to be part of the critically ill condition and its treatment. Second, we *a priori *postulated a difference between CLP-fed and healthy-fed mice, with no difference between CLP-fed and CLP-restricted mice. For the second negative hypothesis, the study may not have been specifically powered.

## Conclusions

Adipogenesis and M2 macrophage accumulation in AT appears to be a hallmark of critical illness, independent of nutritional intake. Accumulation of M2 macrophages is present not only in AT, but also in the liver and lungs suggesting an important global role during illness. Further investigation of the metabolic meaning and underlying triggers of these alterations may shed light on the function of adipose tissue during critical illness and its potential role in the "obesity paradox".

## Key messages

• Adipogenesis and accumulation of tissue M2-macrophages are hallmarks of prolonged critical illness, irrespective of nutritional management.

• M2-macrophages accumulate not only in AT, but also in the liver and lungs.

## Abbreviations

AT: adipose tissue; AU: arbitrary units; BMI: body mass index; C/EBPβ: CCAAT/enhancer binding protein-β; CCL2: chemokine (C-C motif) ligand 2; CI: critical illness; CLP: cecal ligation and puncture; DEXA: dual-energy-X-ray-absorptiometry; FFA: free fatty acids; HSL: hormone sensitive lipase; ICU: intensive care unit; IL-10: Interleukin 10; iNOS: inducible nitric oxide synthase; i.p.: intraperitoneal; IQR: inter-quartile range; i.v.: intravenous; PCNA: proliferating cell nuclear antigen; PN: parenteral nutrition; PPARγ: peroxisome proliferator-activated receptor γ; sc: subcutaneous; TNFα: tumor necrosis factor-α; TPN: total parenteral nutrition.

## Competing interests

The authors declare that they have no competing interests.

## Authors' contributions

MM developed the mice model of sepsis, participated in the design of the study and in data collection, analyzed the results and drafted the manuscript. SVP participated in the molecular gene expression studies and immunostainings. AA participated in the animal experiments. SD participated in the development of the animal model. FG analyzed the images with in-house built computer algorithms. MC and GH participated in the human study. GV participated in its design and coordination, and reviewed the manuscript. LL designed and coordinated the study, participated in the logistics of the animal experiments, data collection, analyzed the results and drafted the manuscript. All authors read and approved the final manuscript for publication.

## References

[B1] Hutagalung R Marques J Kobylka K Zeidan M Kabisch B Brunkhorst F Reinhart K Sakr Y The obesity paradox in surgical intensive care unit patients Intensive Care Med 2011 17 1793 1799 10.1007/s00134-011-2321-2 21818652

[B2] Peake SL Moran JL Ghelani DR Lloyd AJ Walker MJ The effect of obesity on 12-month survival following admission to intensive care: a prospective study Crit Care Med 2006 17 2929 2939 17075374 10.1097/01.CCM.0000248726.75699.B1

[B3] Ray DE Matchett SC Baker K Wasser T Young MJ The effect of body mass index on patient outcomes in a medical ICU Chest 2005 17 2125 2131 10.1378/chest.127.6.2125 15947330

[B4] Tang QQ Lane MD Adipogenesis: from stem cell to adipocyte Annu Rev Biochem 2012 17 715 736 10.1146/annurev-biochem-052110-115718 22463691

[B5] Langouche L Vander Perre S Thiessen S Gunst J Hermans G D'Hoore A Kola B Korbonits M Van den Berghe G Alterations in adipose tissue during critical illness: an adaptive and protective response? Am J Respir Crit Care Med 2010 17 507 516 10.1164/rccm.200909-1395OC 20442437

[B6] Langouche L Marques MB Ingels C Gunst J Derde S Vander Perre S D'Hoore A Van den Berghe G Critical illness induces alternative activation of M2 macrophages in adipose tissue Crit Care 2011 17 R245 10.1186/cc10503 22018099 PMC3334796

[B7] Roberts R Hodson L Dennis AL Neville MJ Humphreys SM Harnden KE Micklem KJ Frayn KN Markers of *de novo *lipogenesis in adipose tissue: associations with small adipocytes and insulin sensitivity in humans Diabetologia 2009 17 882 890 10.1007/s00125-009-1300-4 19252892

[B8] Gordon S Martinez FO Alternative activation of macrophages: mechanism and functions Immunity 2010 17 593 604 10.1016/j.immuni.2010.05.007 20510870

[B9] Curat CA Miranville A Sengenes C Diehl M Tonus C Busse R Bouloumie A From blood monocytes to adipose tissue-resident macrophages: induction of diapedesis by human mature adipocytes Diabetes 2004 17 1285 1292 10.2337/diabetes.53.5.1285 15111498

[B10] Bosco MC Puppo M Blengio F Fraone T Cappello P Giovarelli M Varesio L Monocytes and dendritic cells in a hypoxic environment: spotlights on chemotaxis and migration Immunobiology 2008 17 733 749 10.1016/j.imbio.2008.07.031 18926289

[B11] Hoffstedt J Arner P Hellers G Lonnqvist F Variation in adrenergic regulation of lipolysis between omental and subcutaneous adipocytes from obese and non-obese men J Lipid Res 1997 17 795 804 9144094

[B12] Casaer MP Mesotten D Hermans G Wouters PJ Schetz M Meyfroidt G Van Cromphaut S Ingels C Meersseman P Muller J Vlasselaers D Debaveye Y Desmet L Dubois J Van Assche A Vanderheyden S Wilmer A Van den Berghe G Early versus late parenteral nutrition in critically ill adults N Engl J Med 2011 17 506 517 10.1056/NEJMoa1102662 21714640

[B13] Baker CC Chaudry IH Gaines HO Baue AE Evaluation of factors affecting mortality rate after sepsis in a murine cecal ligation and puncture model Surgery 1983 17 331 335 6879447

[B14] Rittirsch D Huber-Lang MS Flierl MA Ward PA Immunodesign of experimental sepsis by cecal ligation and puncture Nat Protoc 2009 17 31 36 19131954 10.1038/nprot.2008.214 PMC2754226

[B15] Singleton KD Wischmeyer PE Distance of cecum ligated influences mortality, tumor necrosis factor-alpha and interleukin-6 expression following cecal ligation and puncture in the rat Eur Surg Res 2003 17 486 491 10.1159/000073387 14593232

[B16] Hollenberg SM Dumasius A Easington C Colilla SA Neumann A Parrillo JE Characterization of a hyperdynamic murine model of resuscitated sepsis using echocardiography Am J Respir Crit Care Med 2001 17 891 895 10.1164/ajrccm.164.5.2010073 11549551

[B17] Tallarida RJ Cowan A Raffa RB On deriving the dose-effect relation of an unknown second component: an example using buprenorphine preclinical data Drug Alcohol Depend 2010 17 126 129 10.1016/j.drugalcdep.2009.12.014 20061095 PMC3996554

[B18] Langford DJ Bailey AL Chanda ML Drummond TE Echols S Glick S Ingrao J Klassen-Ross T Lacroix-Fralish ML Matsumiya L Sorge RE Sotocinal SG Tabaka JM Wong D van den Maagdenberg AM Ferrari MD Craig KD Mogil JS Coding of facial expressions of pain in the laboratory mouse Nat Methods 2010 17 447 449 10.1038/nmeth.1455 20453868

[B19] Casaer MP Hermans G Wilmer A Van den Berghe G Impact of early parenteral nutrition completing enteral nutrition in adult critically ill patients (EPaNIC trial): a study protocol and statistical analysis plan for a randomized controlled trial Trials 2011 17 21 10.1186/1745-6215-12-21 21261975 PMC3033837

[B20] Mebis L Eerdekens A Güiza F Princen L Derde S Vanwijngaerden YM Vanhorebeek I Darras VM Van den Berghe G Langouche L Contribution of nutritional deficit to the pathogenesis of the nonthyroidal illness syndrome in critical illness: a rabbit model study Endocrinology 2012 17 973 984 10.1210/en.2011-1411 22166982

[B21] Bjorntorp P Effects of age, sex, and clinical conditions on adipose tissue cellularity in man Metabolism 1974 17 1091 1102 10.1016/0026-0495(74)90076-6 4607783

[B22] Langouche L Vanhorebeek I Vlasselaers D Vander Perre S Wouters PJ Skogstrand K Hansen TK Van den Berghe G Intensive insulin therapy protects the endothelium of critically ill patients J Clin Invest 2005 17 2277 2286 10.1172/JCI25385 16075063 PMC1180545

[B23] Sargis RM Johnson DN Choudhury RA Brady MJ Environmental endocrine disruptors promote adipogenesis in the 3T3-L1 cell line through glucocorticoid receptor activation Obesity (Silver Spring) 2010 17 1283 1288 10.1038/oby.2009.419 19927138 PMC3957336

[B24] Chinetti-Gbaguidi G Staels B Macrophage polarization in metabolic disorders: functions and regulation Curr Opin Lipidol 2011 17 365 372 10.1097/MOL.0b013e32834a77b4 21825981 PMC3565956

[B25] Vanhorebeek I Van den Berghe G Hormonal and metabolic strategies to attenuate catabolism in critically ill patients Curr Opin Pharmacol 2004 17 621 628 10.1016/j.coph.2004.07.007 15525554

[B26] Hart DW Wolf SE Herndon DN Chinkes DL Lal SO Obeng MK Beauford RB Mlcak RP Energy expenditure and caloric balance after burn: increased feeding leads to fat rather than lean mass accretion Ann Surg 2002 17 152 161 10.1097/00000658-200201000-00020 11753055 PMC1422407

[B27] Jeevanandam M Young DH Schiller WR Influence of parenteral nutrition on rates of net substrate oxidation in severe trauma patients Crit Care Med 1990 17 467 473 10.1097/00003246-199005000-00001 2109673

[B28] Streat SJ Beddoe AH Hill GL Aggressive nutritional support does not prevent protein loss despite fat gain in septic intensive care patients J Trauma 1987 17 262 266 10.1097/00005373-198703000-00006 3104621

[B29] Cree MG Wolfe RR Postburn trauma insulin resistance and fat metabolism Am J Physiol Endocrinol Metab 2008 17 E1 E9 17957035 10.1152/ajpendo.00562.2007

[B30] Vanhorebeek I Gunst J Derde S Derese I Boussemaere M Guiza F Martinet W Timmermans JP D'Hoore A Wouters PJ Van den Berghe G Insufficient activation of autophagy allows cellular damage to accumulate in critically ill patients J Clin Endocrinol Metab 2011 17 E633 E645 10.1210/jc.2010-2563 21270330

[B31] Ahfeldt T Schinzel RT Lee YK Hendrickson D Kaplan A Lum DH Camahort R Xia F Shay J Rhee EP Clish CB Deo RC Shen T Lau FH Cowley A Mowrer G Al-Siddiqi H Nahrendorf M Musunuru K Gerszten RE Rinn JL Cowan CA Programming human pluripotent stem cells into white and brown adipocytes Nat Cell Biol 2012 17 209 219 10.1038/ncb2411 22246346 PMC3385947

[B32] Khovidhunkit W Memon RA Feingold KR Grunfeld C Infection and inflammation-induced proatherogenic changes of lipoproteins J Infect Dis 2000 17 Suppl 3 S462 S472 10839741 10.1086/315611

[B33] Van den Berghe G Intensive insulin therapy in the ICU--reconciling the evidence Nat Rev Endocrinol 2012 17 374 378 22310851 10.1038/nrendo.2012.14

[B34] Jernas M Palming J Sjoholm K Jennische E Svensson PA Gabrielsson BG Levin M Sjögren A Rudemo M Lystig TC Carlsson B Carlsson LM Lönn M Separation of human adipocytes by size: hypertrophic fat cells display distinct gene expression FASEB J 2006 17 1540 1542 10.1096/fj.05-5678fje 16754744

[B35] Collins S Cao W Robidoux J Learning new tricks from old dogs: beta-adrenergic receptors teach new lessons on firing up adipose tissue metabolism Mol Endocrinol 2004 17 2123 2131 10.1210/me.2004-0193 15243132

[B36] Emorine LJ Marullo S Briend-Sutren MM Patey G Tate K Delavier-Klutchko C Strosberg AD Molecular characterization of the human beta 3-adrenergic receptor Science 1989 17 1118 1121 10.1126/science.2570461 2570461

[B37] Hosogai N Fukuhara A Oshima K Miyata Y Tanaka S Segawa K Furukawa S Tochino Y Komuro R Matsuda M Shimomura I Adipose tissue hypoxia in obesity and its impact on adipocytokine dysregulation Diabetes 2007 17 901 911 10.2337/db06-0911 17395738

[B38] Kvetnansky R Ukropec J Laukova M Manz B Pacak K Vargovic P Stress stimulates production of catecholamines in rat adipocytes Cell Mol Neurobiol 2012 17 801 813 10.1007/s10571-012-9822-6 22402834 PMC3419009

[B39] Vargovic P Ukropec J Laukova M Cleary S Manz B Pacak K Kvetnansky R Adipocytes as a new source of catecholamine production FEBS Lett 2011 17 2279 2284 10.1016/j.febslet.2011.06.001 21689652

[B40] Gordon S Taylor PR Monocyte and macrophage heterogeneity Nat Rev Immunol 2005 17 953 964 10.1038/nri1733 16322748

[B41] Mantovani A Sozzani S Locati M Allavena P Sica A Macrophage polarization: tumor-associated macrophages as a paradigm for polarized M2 mononuclear phagocytes Trends Immunol 2002 17 549 555 10.1016/S1471-4906(02)02302-5 12401408

[B42] Vats D Mukundan L Odegaard JI Zhang L Smith KL Morel CR Wagner RA Greaves DR Murray PJ Chawla A Oxidative metabolism and PGC-1beta attenuate macrophage-mediated inflammation Cell Metab 2006 17 13 24 10.1016/j.cmet.2006.05.011 16814729 PMC1904486

[B43] Prieur X Mok CY Velagapudi VR Nunez V Fuentes L Montaner D Ishikawa K Camacho A Barbarroja N O'Rahilly S Sethi JK Dopazo J Orešič M Ricote M Vidal-Puig A Differential lipid partitioning between adipocytes and tissue macrophages modulates macrophage lipotoxicity and M2/M1 polarization in obese mice Diabetes 2011 17 797 809 10.2337/db10-0705 21266330 PMC3046840

[B44] Vidal-Puig A Unger RH Special issue on lipotoxicity Biochim Biophys Acta 2010 17 207 208 10.1016/j.bbalip.2009.12.010 20045080

[B45] Odegaard JI Ricardo-Gonzalez RR Goforth MH Morel CR Subramanian V Mukundan L Red Eagle A Vats D Brombacher F Ferrante AW Chawla A Macrophage-specific PPARgamma controls alternative activation and improves insulin resistance Nature 2007 17 1116 1120 10.1038/nature05894 17515919 PMC2587297

[B46] Bouhlel MA Derudas B Rigamonti E Dievart R Brozek J Haulon S Zawadzki C Jude B Torpier G Marx N Staels B Chinetti-Gbaguidi G PPARgamma activation primes human monocytes into alternative M2 macrophages with anti-inflammatory properties Cell Metab 2007 17 137 143 10.1016/j.cmet.2007.06.010 17681149

[B47] Shapiro H Lutaty A Ariel A Macrophages, meta-inflammation, and immuno-metabolism ScientificWorldJournal 2011 17 2509 2529 22235182 10.1100/2011/397971 PMC3253544

[B48] Nagy L Szanto A Szatmari I Szeles L Nuclear hormone receptors enable macrophages and dendritic cells to sense their lipid environment and shape their immune response Physiol Rev 2012 17 739 789 10.1152/physrev.00004.2011 22535896

